# Challenges in diagnosis and health care in polycystic ovary syndrome in Canada: a patient view to improve health care

**DOI:** 10.1186/s12905-023-02732-2

**Published:** 2023-11-04

**Authors:** Beate C. Sydora, Michaelann S. Wilke, Maggie McPherson, Sarah Chambers, Mahua Ghosh, Donna F. Vine

**Affiliations:** 1 Metabolic and Cardiovascular Disease Laboratory, University of Alberta, Edmonton, Canada; 2 Division of Endocrinology and Metabolism, Department of Medicine, Faculty of Medicine & Dentistry, University of Alberta, Edmonton, Canada

**Keywords:** Polycystic ovary syndrome, Health care, Health care improvement, PCOS experience, PCOS referral, PCOS follow-up

## Abstract

**Background:**

Polycystic Ovary Syndrome (PCOS) is the most common endocrine-metabolic disorder affecting health and quality of life of those affected across the lifespan. We currently have limited evidence-based data on the experience of those living with PCOS in the health care system including diagnosis, health concerns and disease management. The aim of this study was to assess the perceptions of health status, health care experience and disease management support in those affected by PCOS in Alberta, Canada.

**Methods:**

An online questionnaire was completed via REDCap by individuals self-reporting a diagnosis of PCOS. Question categories included demographics, symptoms of PCOS and time to confirm a diagnosis, follow-up care, health concerns, and information resources. Descriptive statistics were used and thematic analyses was applied to open-response questions.

**Results:**

Responses from 194 participants living in Canada (93% in Alberta) were included. The average age was 34 ± 8 years and BMI was 35 ± 9. Menstrual irregularity was identified in 84% of respondents as the first symptom noticed and the primary reason for seeking a medical consultation. A PCOS diagnosis occurred on average 4.3 years following awareness of first symptoms and required consultation with more than one primary care provider for 57% of respondents. Half (53%) of respondents reported not receiving a referral to specialists for follow-up care and 70% were not informed about long-term health morbidity such as diabetes or cardiovascular disease. Most respondents (82%) did their own research about PCOS using on-line sources, academic literature and advice from peer support. The participant themes from open questions for improving health care included more resources and support, increased and reliable information, better education and training for clinicians, timely diagnosis, prompt referrals to specialists, and generally more compassion and empathy to the challenges faced by those managing their disease.

**Conclusion:**

Our findings highlight the health concerns and challenges in health care for those with PCOS. In Alberta, Canada we have identified major gaps in health care including a timely diagnosis, follow up care and supports, and multidisciplinary care. This evidence-based data can be used to inform development of pathways to improve the health care experience in those affected by PCOS.

**Supplementary Information:**

The online version contains supplementary material available at 10.1186/s12905-023-02732-2.

## Background

Polycystic Ovary Syndrome (PCOS) is the most common endocrine-metabolic disorder affecting 6%–20% of populations [1–4]. PCOS impacts overall health and quality of life across the lifespan from childhood to post-menopause [5–9]. PCOS is associated with increased co-morbidities including obesity, infertility, pregnancy complications, depression and anxiety, metabolic syndrome, diabetes, reproductive cancers and cardiovascular disease [3, 7, 8, 10–13]. The economic health care burden is estimated to be twofold that of individuals without PCOS related to diabetes, cardiovascular disease and gestational complications of hypertension and diabetes [14]. Individuals with PCOS experience significant impacts on their quality of life related to irregular menstrual cycles, heavy menstrual bleeding, infertility, hirsutism, acne, obesity, and cardiometabolic disease risk [3, 8, 15]. In addition, they experience decreased psychological and emotional well-being, anxiety, depression, eating disorders, negative self-image and impaired sexual function [8, 16–19].

The diagnosis of PCOS is based on the presence of two out of the following: clinical and/or biochemical hyperandrogenism, menstrual-ovary dysfunction and/or polycystic ovary morphology, and the exclusion of other endocrine disorders [3]. The need to monitor and track symptoms, and to rule out other endocrine or pathophysiological conditions makes a diagnosis clinically challenging [3]. Another challenge in PCOS diagnosis is the heterogeneity in the presentation of clinical symptoms [20, 21]. Often each clinical symptom and comorbidity is treated separately, which can delay or obscure a diagnosis of PCOS and limit follow up care [3, 22, 23]. Although it can be diagnosed in adolescence, many young individuals may not know they have PCOS or do not receive a diagnosis until they experience infertility [3, 9, 24–29]. Delays in diagnosis may result in a lack of preventative and comprehensive treatment of both symptoms and co-morbidities, further reducing quality of life and increasing risk of morbidity.

Recent studies including surveys, interviews, and focus groups of those with PCOS reveal a high degree of dissatisfaction with health care and the health care system in many countries [2, 28, 30–34]. Dissatisfaction primarily surrounds delayed diagnosis and lack of referral to specialist care, but also lack of information and treatment options. A scoping review of qualitative studies investigating the diagnostic experience of individuals living with PCOS, indicated that the prolonged and complex diagnostic process often leaves patients emotionally drained and can lead to mistrust in the health care system, impacting individual and population health [35]. A lack of information provided to patients at diagnoses may also cause confusion about the cause of symptoms and the disease, generating feelings of guilt and a lack of control [36]. Collectively, these experiences can impact physical and mental health, and overall quality of life [37].

Evidence-based guidelines provide clinicians with recommendations for diagnosis, treatment and management of PCOS [3]. The first-line intervention recommended in PCOS is diet-lifestyle to target body weight and symptom management [3, 38]. However, evidence indicates that the advice and support for diet-lifestyle management is not consistently provided and may not meet the needs of patients [32].

Despite the high prevalence of PCOS, reduced quality of life and increased risk of co-morbidities, the health and health care needs of PCOS patients in different health care settings and geographical locations remains understudied. It is vital to consider the lived experience of those with PCOS in order to understand how the health care system meets their needs and how pathways to better health care can be improved. We currently have a paucity of evidence on the barriers and challenges faced by individuals with PCOS in the self-management of their disease and in the health care system in Alberta and Canada. Our study aimed to gather evidence on the barriers and challenges in those with PCOS in managing their disease in the health care system in Canada. We assessed perceptions of health status, health care experience and disease management support in those affected by PCOS in Alberta, Canada. Understanding the health status and health care experience of those affected by PCOS can help guide development of pathways to care that ultimately improve health and quality of life in this population.

## Methods

### Study design

We conducted a survey to evaluate the health concerns and health care experience in those affected by PCOS. The survey methodology followed the recommendations of the Checklist for Reporting Results of Internet E-Surveys (CHERRIES) to ensure that this study was conducted in a reliable, reproducible, and transparent manner [39]. The survey was created using REDCap (Research Electronic Data Capture) [40], a web-based application used for electronic data capture, hosted and supported by the Women and Children’s Health Research Institute (WCHRI) at the University of Alberta. It was adapted from a previously published PCOS questionnaire including a range of questions on health care issues and experiences [2]. Our extended questionnaire was pilot-tested in PCOS patient-partners (*n* = 5) and health professionals who care for those with PCOS (Endocrinologist, Obstetrician-gynaecologist, General Practitioner, Registered Dietitian, Primary Care Nurse) (*n* = 5) to test adequacy, appropriateness, thoroughness, clarity and understandability of the questions and multiple-choice options relevant to the Alberta Health Care system. The questionnaire included 72 questions in 5 sections; 4 of which are subject of this paper: 1) demographics, 2) PCOS symptoms and diagnosis, 3) follow-up care, 4) overall health concerns and information resources.

### Confidentiality and ethics consideration

Participation in the survey was voluntary and completely anonymous; no identifying information was retrieved. Potential participants were informed that completion would take about 60 min and submission of the questionnaire constituted consent to participate in the study. Participants could choose not to answer questions if they felt uncomfortable to do so. In addition, participants could withdraw at any time from the survey by closing the link. Given the anonymous nature of the survey, once responses were submitted, it was no longer possible to withdraw from the study. The study was approved by the University of Alberta Health Research Ethics Board (Pro0005542).

### Participants and setting

The survey was undertaken in individuals in Alberta, Canada who self-identified to have PCOS. It was openly distributed by link through social media, various patient registries and research-focused websites, including: PCOS-Together website and the Alberta SPOR SUPPORT Unit (AbSPORU) registry [41–43]. Responses were collected online into the REDCap database during January 2021 to June 2022. One duplicate entry was identified and was removed from analyses.

### Data and statistical analyses

Data was exported from REDCap to Microsoft Excel for analysis. For the current study we focused on the survey data that were relevant to provide insight into the experience of PCOS symptoms, diagnosis and the individual health care journeys through the health care system. Descriptive statistics were applied to categorical and continuous data. Categorical data was expressed as number and percentage of responses to each question. Continuous data was reported as mean ± standard deviation (SD). Patient-reported height and weight were used to calculate BMI, and age at the time of survey completion was calculated based on year of birth. Open questions were analyzed using qualitative content analysis and inductive coding methodology [44]. Textual data from answers was organized into codes and integrated into broader categories to generate themes. Themes were reviewed and agreed upon by three independent reviewers. Themes were supported by adding examples of quotes in response to the open-ended questions.

## Results

### Respondent characteristics

Survey respondent demographic characteristics are summarized in Table 1. Of the total 222 respondents, 198 completed the survey and 194 responses were included in the results based on residence in Canada. Respondents mostly resided in the province of Alberta, 93%, at the time of survey completion. The majority (90.7%) of respondents were born in Canada and 9.8% were immigrants to Canada. While 187 (96.4%) stated to have received a PCOS diagnosis, 7 (3.6%) suspected to have PCOS, but did not receive a clinical diagnosis. All respondents identified as of the female gender, except for one non-binary respondent. In addition to identifying as a female, 4.6% disclosed a LGBTQ + identity. Most of the participants (84.5%) were Caucasian/White and 13.9% self-identified as a member of a minority group. A proportion (18.6%) reported to have a disability. The average of the self-reported height and weight was 166 ± 7 cm and 96 ± 27 kg, with an average body mass index (BMI) of 35 ± 9 kg/m^2^ (Table 1). The calculated BMI for the majority of participants (77.5%) met the criterial cut-off for obesity with a BMI > 30, while 7.7% were overweight with a calculated BMI between 25 and 30 kg/m^2^, and 14.8% had a BMI < 25 kg/m^2^.

**Table 1:** Demographic and anthropometric characteristics of survey respondents

Respondent characteristics for *N* = 194		N [%]
***Residence***	Alberta	180 [92.8]
	Outside of Alberta in Canada^a^	15 [7.7]
***Birthplace***	Born in Canada	176 [90.7]
	Born outside of Canada	19 [9.8]
***Sex and Gender identification***	Female	193 [99.5]
	LGBTQ +	9 [4.6]
	Non-binary/gender fluid	1 [0.5]
***Ethnicity***	Caucasian/White	164 [84.5]
	Asian	14 [7.2]
	African/Black	6 [3.1]
	Indigenous/First Nations	4 [2.1]
	Other	6 [3.1]
***Self-identified member of minority group***	No	157 [80.9]
	Yes	27 [13.9]
	Prefer not to answer	10 [5.2]
***Disability***	No	147 [75.8]
	Yes	36 [18.6]
	Prefer not to answer	11 [5.7]
	**range**	**[mean ± SD]**
***Age*** (years)	21 – 79	33.98 ± 8.36
***Height*** (cm)^b^	152 – 185	165.83 ± 6.95
***Body weight*** (kg)^b^	34.0 – 168.0	95.93 ± 26.90
***Body mass index calculated*** (kg/m^2^)	14.6 – 61.6	34.90 ± 9.38

^a^includes 6 from Ontario, 3 from British Columbia, 3 from Atlantic Canada and one each from Saskatchewan, Quebec and NWT/Yukon/Nunavut

^b^height was not reported for 6 respondents and weight was not reported for 3 respondents; BMI could not be calculated for 9 respondents

Respondents’ socioeconomic attributes are outlined in Supplementary Table 1 (Supplementary Table S1). Most respondents (76.8%) had education after high school including a certificate, graduate, or postgraduate degree. The majority of the participants were employed and about half (45.9%) reported an annual household income between 30,000 and 80,000 CAD. While 60% of respondents did not have children, 36.6% had between 1 and 3 children and 6 reported having 4 or more children. The majority of respondents, 75.8%, lived with a partner with or without dependents and 45.9% lived in very large population urban centres, defined as a centre with a population of 500,000 or more (Supplementary Table S1).

### PCOS-related health care at diagnosis

#### Symptom onset and age of diagnosis

In 58.2% of respondents, first PCOS-related symptoms were noticed in the childhood-adolescent years between 10–18 years of age, whilst 31.4% of respondents became aware of their symptoms at age 19–29 years, and 6.7% at > 29 years of age. No-one reported to be older than 39 years at the time of first symptom awareness. The first symptoms noticed in 83.5% of respondents were menstrual irregularities, excess hair growth (50.5%), body weight gain (49.5%), and acne (47.9%) (Table 2). Infertility was among the first symptoms noticed for 18% of respondents. Correspondingly, menstrual irregularities were also the main reason for seeking medical help in 74.2% of respondents. The average age of those seeking medical help based on symptoms was 20 years, and ranged from 10 to 42 years of age.

**Table 2:** Survey respondents’ experience of symptom onset and diagnosis

**Symptom experience**		**N [%]**
***Symptoms first noticed***^a^	Menstrual irregularities	162 (83.5)
	Excess hair growth	98 (50.5)
	Body weight gain	96 (49.5)
	Acne	93 (47.9)
	Infertility	35 (18.0)
	Other symptoms^b^	39 (20.6)
***Symptoms that led to seek medical help***	Menstrual irregularities	144 (74.2)
	Body weight gain	63 (32.5)
	Excess hair growth	56 (28.9)
	Acne	51 (26.23)
	Infertility	53 (27.3)
	Other symptoms^c^	24 (12.4)
	Other reasons outside of symptoms^d^	8 (4.1)
**Time frames [years]**	**range**	**[mean ± SD]**
***Age at first seeking medical help***	10–42	19.98 ± 6.14
***Age at diagnosis***^e^	13–36	24.01 ± 6.12
***Time between first seeking help and diagnosis***	< 1–20	4.25 ± 5.86
**Diagnosis**		**N [%]**
***Clinician who made PCOS diagnosis***	Family doctor	98 (50.5)
	Referred specialist	73 (37.6)
	More than one family doctor or referred specialist^f^	110 (56.7)
***Information about PCOS-related potential long-term health complications***	Received information at diagnosis	47 [24.2]
	Did not receive information at diagnosis	135 [69.6]
	Not reported	12 [6.2]
**Recommendations and treatment**	**Themes from open questions**^g^	**N [%]**
***Information provided***	Pregnancy/fertility-related information	20 [10.3]
	Verbal information including diabetes risk	7 [3.6]
	Verbal information including high blood pressure	3 [1.5]
	Verbal information including mental issues	1 [0.5]
	PCOS information printout/website	4 [2.1]
***Recommendation and advice***	Loose weight	28 [14.4]
	Exercise	3 [1.5]
	No to worry until wish for babies	9 [4.6]
	Consult naturopath	1 [0.5]
***Prescription***	Birth control	35 [18.0]
	Metformin	16 [8.2]
	Letrozole	2 [1.0]
	Spironolactone	2 [1.0]
	Clomid	1 [0.5]
	Ozempic	1 [0.5]

^a^Respondents could choose more than on answer

^b^Include fatigue, severe cramps, pain, migraines, hair loss

^c^Include fatigue, severe menstrual bleeding, severe lower abdominal pain, hair loss

^d^Include IUD check up, pap test, physical exam, ultra sound for pain

^e^*n* = 5 respondents reported that their have not received a definite diagnosis of PCOS

^f^may include one or more than one Family doctor, Endocrinologist, Gynecologist, Obstetrician, Fertility Specialist, Naturopath or alternative medicine practitioner

^g^more than one information of recommendation could have been provided

The average age at PCOS diagnosis was 24 years with an age range from 13 to 36 years; 5 women had not received a definite PCOS diagnosis including a respondent aged 42 years (Table 2). For those who had received a clinical diagnosis, the time between first seeking medical help for PCOS-like symptoms and when a PCOS diagnosis was made averaged 4.3 years, and ranged from less than 6 months for 38.1% of respondents to over 2 years for 31.4% respondents. For half of the respondents a family physician made the PCOS diagnosis and in 37.6% of patients, diagnosis was made by a specialist (endocrinologist or obstetrics-gynecology clinician) following referral. In 56.7% of respondents a diagnosis was made following more than one visit to a family physician, or one or more specialists (Table 2). Living in or near a large urban population centre at the time of diagnosis did not appear to affect diagnostic services. While 40.7% of respondents lived in a large city (population density of 500,000 residents or more) at the time of the diagnosis, there was no statistically significant difference in who made their PCOS diagnosis (family doctor versus referred specialist versus more than one clinician) when compared to those living in smaller urban or rural population centres (*p* > 0.05 for all chi square statistical cross analysis).

#### Information and resources at diagnosis

Only 24.2% of respondents received information about long-term health complications in PCOS at the time of diagnosis, of which 10.3% who were informed about pregnancy/fertility-related issues. Many respondents, 69.6%, did not receive adequate information on common co-morbidities in PCOS such as overweight-obesity, cardiometabolic risk factors, diabetes and cardiovascular disease at the time of diagnosis. Very few respondents reported having received any general information about the PCOS condition or possible health risks either verbally or from resources (handout /websites) (Table 2). When asked to report on advice and resources given to them at the time of their diagnosis, 16% of respondents were given recommendations on body weight, diet and lifestyle, and these were primarily focussed on reducing body weight and exercise. Respondents (4.6%) were advised not to be concerned about their PCOS symptoms until they were planning to conceive (Table 2).

#### Prescribed treatments at diagnosis

Only about one third of respondents (29.2%) reported receiving prescriptions to address symptoms at the time of their diagnosis. The most common prescription was the oral contraceptive at 18%, followed by metformin for 8.2% of respondents. Letrozole, Spironolactone, Clomid and Ozempic were also prescribed (Table 2).

### Co-morbidities, symptoms and follow-up health care for management

#### Co-morbidities and symptom concerns

**Fig. 1:**
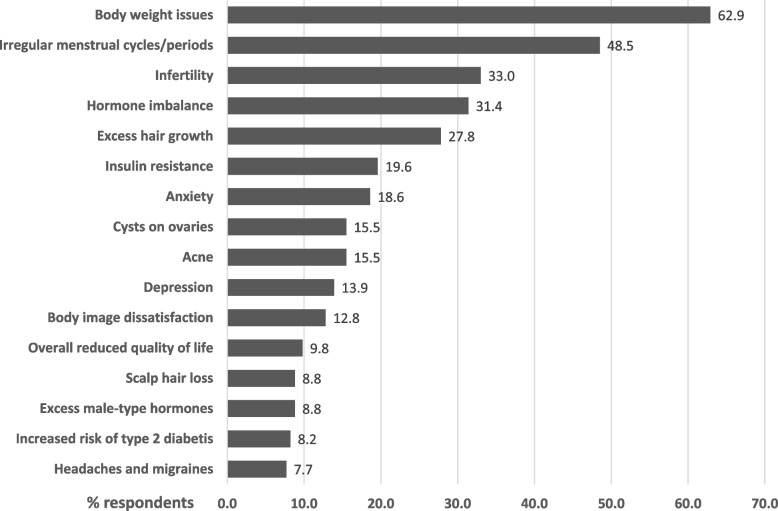
Most important PCOS-related symptoms and health concerns in respondents

A major co-morbidity was increased body weight, experienced by 62.4% of respondents. Other comorbidities included dyslipidemia, metabolic syndrome and diabetes (Table 3). PCOS-related symptoms and health concerns that ranked most significant to respondents are shown in Fig. 1. Body weight was one of the four most important health concerns (62.9%), followed by irregular menses (48.5%), infertility (33.0%), hormone imbalance (31.4%), and excess hair growth (27.8%). Ovarian cysts, acne and hair loss impacted 15.5, 15.5 and 8.8% of respondents, respectively. Insulin resistance and risk of diabetes concerned 19.6 and 8.2% of respondents, respectively. Mental health issues included anxiety and depression, which were of concern in approximately one third of respondents. Health concerns also included premenstrual syndrome, increased risk for cardiovascular disease, ovarian and endometrial cancer, and sleep apnea in less than 7% of respondents.

**Table 3:** Co-morbidities and follow-up health care in those with PCOS

Follow-up and continuous care		N [%]
***Diagnosis of comorbid health issues***	Overweight and obesity	121 [62.4]
	High LDL cholesterol	30 [15.5]
	High Glucose (pre-diabetes)	26 [13.4]
	High triglyceride	16 [8.2]
	Metabolic Syndrome	12 [6.2]
	Type 2 Diabetes	6 [3.1]
	Low HDL cholesterol	4 [2.1]
	Heart disease	1 [0.5]
***Referral to other health professionals for FU***	Did not receive referral	103 [53.1]
	Gynecologist	41 [21.1]
	Endocrinologist	21 [10.8]
	Dietician	20 [10.3]
	Exercise Specialist	3 [1.5]
	Psychologist/Psychiatrist	2 [1.0]
	Other	11 [5.7]
	Fertility Specialist	6 [3.1]
	Dermatologist	3 [1.5]
	Weight loss clinic	1 [0.5]
	Naturopath	1 [0.5]
***Clinician or Health professional seen for regular PCOS management***	Did not see anyone regularly	92 [47.4]
	Family Doctor	56 [28.9]
	Gynecologist	20 [10.3]
	Endocrinologist	10 [5.2]
	Registered Dietician	3 [1.5]
	Exercise Specialist	1 [0.5]
	Psychologist/Psychiatrist	1 [0.5]
	Dermatologist	1 [0.5]
	Other Specialist/Health Professional	14 [7.2]
	Naturopath	8 [4.1]
	Internist	1 [0.5]
	Acupuncture	1 [0.5]
	Laser Hair removal	1 [0.5]
***Part of Primary Care Network***	Yes	72 [37.1]
	No	50 [25.8]
	Don’t know	54 [27.8]
***Is care received adequate***	Yes	71 [36.6]
	No	90 [46.4]

#### Health care referral and management

Despite many of these health concerns requiring medical management and treatment, only about half of respondents were provided a referral to other specialized health care professionals following diagnosis. In those respondents that did receive a referral, 21.1% were referred to a gynecologist, 10.8% to an endocrinologist, 10.3% to a dietician, 3.1% to a fertility specialist and 1.5% to a dermatologist (Table 3). Only 1% of respondents were referred to a psychologist or psychiatrist, yet 35.6% reported mental health as a concern. Nearly half of respondents (47.4%) stated that they were not having regular follow up care with a clinician or health care professional to help manage PCOS symptoms. Only 28.9% visited their family physician regularly, and 10.3 and 5.2% indicated that they have regular visits to a gynecologist and endocrinologist, respectively. Body weight, hair-skin and mental health concerns were reported as major health concerns, however many respondents did not visit health professionals such as dieticians, exercise specialists, dermatologists, behaviour therapists or psychologists/psychiatrists to manage these conditions (Table 3). One third of respondents were registered with a Primary Care Network (PCN), which helps support and enable patient access to a multidisciplinary health care team. Approximately half of respondents (46.4%) indicated that their health care was inadequate compared to 36.6%, who reported to have received adequate health care (Table 3). Respondents rated a very low satisfaction with PCOS-related follow up health care and this was reflected in an overall median score of 10 on a scale from 0 (very dissatisfied) to 100 (very satisfied).

#### Prescribed medication at follow-ups

Metformin and combined estrogen/progestin oral contraceptives were the most often prescribed medications in follow-up clinic visits at 44.3% and 39.7%, respectively. Other prescribed medications included progestin-only oral contraceptives (16.0%), non-hormonal contraceptives (18.6%), spironolactone (12.4%), and other anti-androgen and fertility treatments (13.9%). Finasteride to treat hair loss, Accutane to treat acne and Candesartan/Amlodipine to treat heart-related problems were reported by one respondent. Prescribed medicinal treatment of symptoms was reported to be effective to varying degrees. For example, 2 respondents reported that Letrozole was successful in treating symptoms out of 8 respondents receiving Letrozole prescription; the rate of successful treatment for Clomid, Ozempic and Inositol was 2/3, 1/2, and 3/5, respectively; Ovasitol, Finistrate, and Accutane were found effective by all who received a prescription for these medications. In summary, fifteen respondents out of 25 reported that the prescribed medicine was successful in the treatment of the specific symptom it was prescribed for.

### Open statements on information and resources at diagnosis and follow up

Comments on information and resources provided were explored in open questions (Table 4). Of the survey respondents, 62% provided answers with regard to information received at the time of diagnosis and 59% provided feedback related to follow-up care.

**Table 4:** Open comments on information and resources provided by respondents

Themes: information and resources provided at the time of diagnosis [*N* = 121]	N [%]
***insufficient information***	72 [59.5]
◦ “There really wasn't a lot given to me except your typical PCOS lose weight and it frustrated me so I went and researched myself and have restored my menstrual cycle naturally” ◦ “Basically, wasn't given anything. I wasn't actively trying to conceive yet so all my other symptoms and concerns were dismissed.”	
***lack of expert knowledge***	20 [16.5]
◦ “Only there is a weird idea about how its just a women thing. Most of the doctor's I have seen would not deal with it due to it being a woman related problem.” ◦ “I wonder how well-educated male doctors are about female fertility and how these issues affect more than just our ability to reproduce.”	
***no follow-up or referral***	13 [10.7]
◦ “He refuses to listen to my request for a referral.” ◦ “Doctors should give information or refer to a PCOS specialist.”	
***lack of treatment options***	12 [9.9]
◦ “Just nutrition. No other evaluations were completed by the gyno except sending me to a nutritionist and advising me to lose weight” ◦ “Much of the focus of treatment was in the future when trying to have kids only, not lifestyle management for preventative health.”	
***lack of adequate health care***	11 [9.1]
◦ “It was very little and felt like a death sentence in terms of getting diabetes, having to be on the pill for life and would struggle with fertility and would need fertility supports to get pregnant. It was discouraging that the only options I was given was to lose weight and take prescribed medication. No one tried to treat the root problem” ◦ “The Doctor just told me that they could drain the cysts but they would keep coming back.”	
***insufficient resources***	10 [8.3]
◦ “What resources? Or information? I was given nothing. And this was from a fertility specialist who knew I was trying to get pregnant. “ ◦ “Doctors did not know much to be able to provide information or resources.”	
***lack of mental health care***	5 [4.1]
◦ “It's not just the medical condition that's a factor. There's also mental health to consider, primarily depression and anxiety. Depression, from personal experience, can hinder the want to lead a healthy life.” ◦ “Being young and hearing having kids would be extremely difficult without help was extremely mentally challenging. I was put on anti depressants shortly after that. I wish I had more in-depth information then, and now I wish I had access to a good dietician to help with the weight lose issues that come with PCOS.”	
***no complaints, received adequate care***	4 [3.3]
**Themes: information and resources provided by health care professionals at follow-up [*N* = 116]**	
***lack of support***	53 [45.7]
◦ “I think more physicians should be taught about reproductive issues and that each individual deals with pain and issues differently so they shouldn't be judged based on pain tolerance or to say it's "all in your head" or "it's just what women have to deal with.” ◦ “I had doctors who didn't believe me. I have fertility specialist that laughed me out of their office. I feel it's pretty difficult when dealing with PCOS I have managed to completely ostracize myself in some ways because of having to go through some of the hurdles without any supports.”	
***insufficient information***	42 [36.2]
◦ “I did not receive enough information to understand the different options. I started taking birth control but I am not sure what would be my other options, or how I could manage the symptoms without birth control.” ◦ “I am craving information and feel like I have been hunting for answers for years. Still don't feel grounded in a treatment plan.”	
***insufficient follow-up care***	39 [33.6]
◦ “They offer no form of treatment. They just expect you to live with it and deal with on your own.” ◦ “After having my first child and being monitored by an OB there was no follow up on care or living with PCOS”	
***lack of resources***	12 [10.3]
◦ “I received zero resourses or information from any healthcare providers” ◦ “My GP never provided me any resources. The Gynecologist I saw twice, once to do a physical assessment and once to review my ultrasound results, never gave me any resources.”	
***lack of referral***	5 [4.3]
◦ “There were no referrals for more information or treatment options provided” ◦ “I do wish I could be referred to a dietician or naturopath perhaps to see what more could be done, but currently that doesn't seem to be an option by my doctor.”	
***no comments***	5 [4.3]

## Diagnosis

Over a third of respondents (59.5%) felt that they had received insufficient information at the time of their diagnosis to prepare them for their health journey with PCOS. Respondents stated concerns about lack of expert knowledge at their initial clinic visit (16.5%) and 10.7% commented that they were not adequately provided with referrals and/or follow-up care appointments. Other comments included lack of treatment options, adequate care and specific mental health care, insufficient resources to help manage their disease and a delayed start of treatments. Only 3.3% of respondents expressed no complaints or reported that they had received adequate care at the time of their diagnosis (Table 4).

## Follow up

The comments on information and resources provided during follow-up care included lack of supports, resources and referrals, and insufficient information and follow-up care (Table 4). On a scale from 0 (inadequate) to 100 (adequate), survey respondents rated the level of information and resources provided to them with a median score of 13, indicating a very low level of adequate information and resources provided by a clinician or the health care system to help manage their disease. Due to the perceived lack of information on their PCOS-related health issues, 82% of respondents commented that they had to do their own research to find information about their disease. The primary sources of information included internet searches (68.6%) and social media/web sites (40.7%). One quarter of respondents (25.8%) reported getting information from books, others from the academic literature (3.1%). Peer support groups were consulted by 12.4%. Less than 10% of respondents sought self-initiated advice from naturopaths and other specialists such as a dietician and endocrinologist.

### Respondent suggestions for improved support and health care for PCOS

Survey questions were designed to explore how respondents would improve their health care, the health care system and how patients with PCOS could be supported. More than 70% of respondents suggested ‘better access to health professionals’ and to ‘provide more educational material’ for PCOS. Access to a registered dietician and educational material on nutrition were ranked as high priorities (Table 5). Respondents also suggested a PCOS information website (61.9%), inclusion in research studies (60.8%), patient education forums (46.9%) and regular updates on PCOS health information (38.7%). In open comments, one third of respondents suggested provision of more resources and supports, and improved information and treatment options. Clinician training (25.5%), prompt referrals to specialists (19.0%) and a call for more compassion and understanding (16.8%) emerged as themes in open-comments (Table 6).

**Table 5:** Preferred and suggested support and resources for those with PCOS

Preferred and Suggested Resources and Support (from drop-down options)^a^	*N* = 194 N [%]
• Better access to health professionals	150 [77.3]
◦ Registered Dietician	134 [69.1]
◦ Psychologist	111 [57.2]
◦ Exercise Specialist	110 [56.7]
◦ Other	46 [23.7]
• Provide more educational materials	143 [73.3]
◦ Nutrition	138 [71.1]
◦ Exercise	120 [61.9]
◦ Medications	120 [61.9]
◦ Health outcomes	126 [64.9]
◦ Other	12 [6.2]
• Maintain a PCOS information website	120 [61.9]
• Involve research participants in current research design, recruitment and management	118 [60.8]
• Support and present at patient forums and workshops	91 [46.9]
• Send a regular update email with information on PCOS	75 [38.7]
• Other	14 [7.2]

^a^answers included several choices

**Table 6:** Recommendations and advice

**Themes: Recommendations for health care improvement for women with PCOS [*N* = 137]**^a^	**N [%]**
***more resources, more support***	49 [35.8]
◦ “Mayby give more resources instead of leaving the patient to figure it out saying just lose the weight doesn’t help.” ◦ “More doctors at women's clinics that can offer resources and help.”	
***better information, more options***	44 [32.1]
◦ “Provide more information regarding PCOS Additional screening / testing for future complications of PCOS (diabetes, heart problems, pelvic ultrasound, etc.).” ◦ “Give patients the info right there and then. Follow up.”	
***educate/train clinicians***	35 [25.5]
◦ “More doctors need to know about PCOS! They need to know what blood work needs to be done, from what I've seen, they don't.” ◦ “Maybe more training and compassion from primary care physicians so I don't want to avoid going.”	
***prompt referral to specialists***	26 [19.0]
◦ “I think that there should be faster referrals to OB's or GYN'S for any reproductive issue.” ◦ “Referrals to a natural path or dietitian would be helpful. We are left to navigate this alone.”	
***more compassion and understanding***	23 [16.8]
◦ “Often times I've felt neglected and alone with my condition, doctors didn't take me seriously or allow me to believe that not having my period isn't normal.” ◦ “Treat women as more than child-bearers. PCOS affects more than just ovaries. It also affects our mental health.”	
***more pain management***	3 [2.2]
◦ “Listen better to patients who seek help for severe pain.” ◦ “Take our pain seriously. Help us find solutions and not band aid.”	
***no comments***	3 [2.2]
**Themes: Advice for young women with PCOS in Alberta [*N* = 136]**^**a**^	
***be your own advocate, do your own research***	71 [52.2]
◦ “Advocate for yourself, don't be afraid to ask questions and don't let doctors invalid or minimize your symptoms.” ◦ “Do you research before going to an appointment. Stick up for what you want and the path you want to go.”	
***ask questions, get information, more options***	38 [27.9]
◦ “Go see a gynaecologist right away and ask questions and what can be done for it.” ◦ “Ask questions! Seek help for fertility that goes to the root of the issue.”	
***see multiple physicians or specialists, get 2nd opinion***	32 [23.5]
◦ “Find a physician / dietician / therapist, etc. that specializes with PCOS patients.” ◦ “Always do your own research and get more than one opinion on things if you still feel unsure”	
***ask for referral***	20 [14.7]
◦ “If you don't feel heard ask for a new referral, try a naturopath osteopath or even acupuncture it won't damage and it might help.” ◦ “Ask her family doctor to refer her to any specialist she may needs, especially for mental health as it can be an overwhelming diagnosis.”	
***find someone (clinician/support group) who listens***	17 [12.5]
◦ “I would tell her to reach out to local groups of women that have PCOS I would ask her to find a proper physician that will recognize PCOS is a problem and knows a little bit about it and is open to working with them they may not know everything about it but they're willing to send referrals and find different ways to help them find success.” ◦ “Find a friend or hire a coach, nutritionist or dietician who has been through it before and will help you navigate. Someone who can tell you where to start and show you how to design a lifestyle you can keep up for life that doesn't deprive you or cause/worsen eating disorders.”	
***maintain a healthy lifestyle, listen to your body***	13 [9.6]
◦ “Watch what you eat and stay physically healthy.” ◦ “Concentrate on staying healthy, without shame.”	
***don't know where to get help, no advice***	6 [4.4]

^a^answers could be included in several themes

The advice, the respondents would provide to a newly diagnosed individual with PCOS to help guide them through the health care system in Alberta, included self-help initiatives such as self-advocacy, self-research and asking for help (52.2%). Asking questions to get more information and receive more care options was advised by 27.9%, and 23.5% suggested getting a second opinion from another physician or specialist. Respondents (14.7%) also considered it important to ask for a referral to a specialist, while 12.5% highlighted the need for social support, finding someone who would listen or joining PCOS support groups. Maintaining a healthy lifestyle such as eating healthy food and listening to your body emerged as a theme in 9.6% of respondents (Table 6).

## Discussion

The result of our survey in those affected by PCOS highlights the health concerns and challenges in the health care system from diagnosis to follow-up and continuous long-term care, and quest for resources to help manage their disease. The main challenges faced by those affected by PCOS were found to be delays in diagnosis, insufficient management and treatment options, and a lack of information on potential long-term morbidities and preventative health care. Perceived barriers in the health care system included a lack of multidisciplinary care, lack of knowledge and empathy from health care providers and delays or no referral to specialists such as endocrinologists, dieticians, and psychologists. The outcomes of this survey reflect the health care experience of those with PCOS in the province of Alberta, which has broad similarities with other Canadian provincial health care systems but these may also differ in specific health policies, initiatives and resource allocation [45]. Our findings reinforce other findings in Canada and as well as other countries related to the health care experience of those with PCOS [2, 30–32, 34, 46]. This suggests health care challenges of those affected by PCOS are not regionally specific but are universal to a disease that is largely understudied and under-recognized as a major public health concern.

### Diagnosis

Our study participants reported a protracted PCOS diagnosis that occurred at an average age of 23 years and took on average greater than 4 years following the first physician consultation for PCOS-related symptoms. The delay in diagnosis may be attributable to insufficient medical training of physicians in women’s health issues related to menstruation and PCOS specifically, and a lack of referral to endocrine and women’s health specialists [33, 47]. Our data is consistent with other reports of a protracted diagnosis of PCOS taking greater than two years, and associated with multiple visits to health professionals and examination by three or more physicians before a diagnosis [2, 30, 48]. It is not clear whether these diagnostic delays are associated with a lack of physician education and training about PCOS and diagnostic criteria. For instance, there were 35 respondents whose diagnoses took 10 years or more from the first physician visit concerning symptoms. Of those, 10 respondents questioned their physician’s knowledge of PCOS and stated that they thought the physician dismissed their symptoms. A study conducted in Australia and North America revealed physicians had significant gaps in the knowledge of PCOS, diagnostic criteria and related comorbidities [49, 50]. The importance of a timely and early diagnosis in a young individual affected by PCOS has been determined as necessary for effective intervention and management to prevent further deterioration in mental and physical health [3, 37].

### Lack of information at diagnosis

In addition to a protracted diagnosis, over 70% of respondents reported a lack of information about PCOS at the time of diagnosis. This finding is consistent with other studies, using focus groups and surveys, that report information provided at diagnosis was not sufficient to allow for improved knowledge and effective self-management of PCOS symptoms [2, 46, 48]. Information and resources on basic anatomy of the reproductive system, PCOS symptoms, potential treatments for a range of symptoms and other health risks are important to provide at diagnosis to enable patients to self-manage and improve knowledge of their disease, as recommended in the international guidelines [3]. A PCOS diagnosis can lead to self-imposed diet-lifestyle changes or treatments that create additional anxiety and stress; therefore, appropriate personalized health communication of information about PCOS and resources at the time of diagnosis are important [51]. A sensitive-personalized approach to communication of a diagnosis and information about PCOS has been recommended to avoid unnecessary stress and anxiety associated with a fear of infertility and other health risks when receiving a PCOS diagnosis [3, 52]. For example, a fear of infertility or an expectation of infertility following a PCOS diagnosis may lead to individuals with PCOS not using contraception resulting in unplanned pregnancies [51].

### Referrals and follow-up in health care management

Many respondents reported that at the time of diagnosis they were not provided with a follow up treatment plan, and follow up health care was dependent on their immediate to short-term plans to have children. If they were not planning to become pregnant in the short term, the need for follow-up care was dismissed and delayed. Therefore, these individuals did not receive the necessary care required to assess cardiometabolic risk factors and risk of serious co-morbidities such as Type 2 Diabetes and cardiovascular disease, which are recognized in the international guidelines as important to address in management of PCOS [3, 53, 54]. Respondents stated follow-up health care should not be restricted to periods related to childbearing. These findings were consistent with a Canadian study reporting that patients were dismissed and deferred by their physician to follow-up care until planning to become pregnant [47]. Those with PCOS have an increased risk of pregnancy complications such as gestational diabetes and pre-eclampsia, and post-partum risk for diabetes and cardiovascular disease. Therefore, early intervention to target cardiometabolic risk factors following a diagnosis of PCOS is strongly recommended [3, 55, 56].

Of note, 57% of respondents considered it important receive or have better access to mental health services such as psychologists. However, only two respondents were referred to a psychologist/psychiatrist in a follow-up visit and only one respondent was seeing a psychologist/psychiatrist regularly at the time of the survey. This is concerning given the established evidence of increased depression, anxiety, eating disorders and other mental illnesses in those affected with PCOS [3, 8, 57–59]. Prime symptoms that appear to be associated with depression and anxiety include acne, hirsutism, obesity and infertility. These psychiatric morbidities greatly impact quality of life in those with PCOS [3, 60]. In addition, depression and anxiety can impede an individual’s ability to follow challenging recommendations such as diet-lifestyle interventions to target body weight. Depression and anxiety in those affected by PCOS are also risk factors for eating disorders [8, 18, 61–63]. Thus, routine screening for mental illnesses including anxiety, depression and eating disorders at the time of PCOS diagnosis or during follow-up care and referral to psychiatric counselling is recommended for those with PCOS [3, 8, 57, 58].

Lack of follow-up health care management by physicians and referral to other allied health professionals was not the only concern in those with PCOS. Respondents noted that PCOS symptoms may be dismissed and not taken seriously. Other barriers to PCOS symptom management also include time and/or geographical restrictions to be able to access follow-up health care from specialists [64]. In addition, those affected by PCOS may not internalize all information given to them at diagnosis, and may experience low motivation and compliance to follow diet-lifestyle recommendations when they learn their disease has no cure [64].

### Clinical treatment options and medical prescriptions

Therapeutic options for PCOS symptom management vary depending on symptom presentation, and the priorities and needs of the patient [3]. Menstrual dysfunction-acyclicity, hirsutism, acne, alopecia, endometriosis, and fertility concerns can be treated with a range of interventions and pharmaceuticals including oral contraceptives and anti-androgens [3, 20]. At diagnosis, less than 25% of respondents indicated that they were provided prescriptions for treatment of PCOS symptoms. Of these, the highest number of prescriptions (18%) were for oral contraceptives, including combined estrogen/progestin and progestin-only contraceptives and a variety of non-oral hormonal contraceptives. Oral contraceptives are commonly prescribed for those not considering becoming pregnant to treat the primary concerns of menstrual irregularities and hyperandrogenism [3, 65, 66]. Metformin was prescribed in 8% and 44% of respondents at diagnosis and at follow-up clinic visits, respectively, to target insulin resistance and pre-diabetes risk, androgen production, cardiometabolic risk factors and reproductive concerns [67–70]. Respondents also reported pharmaceutical treatment for specific symptoms such as Spironolactone for hirsutism, Finasteride for alopecia, and Accutane for treatment of acne. Respondents reported that they felt there were limited treatment options for PCOS symptoms and oral contraceptives were offered as a first-line treatment, however this was not necessarily a preferred treatment option by respondents.

### Resources and information

A general lack of information and resources was identified by many respondents as a barrier in effective management of PCOS at diagnosis and follow up. This included primarily a lack of verbal information or informative material for patient education and management options, access or referral to allied health care professionals, specialists and peer support groups. There was also the sentiment that general practitioners were not sufficiently educated about PCOS to satisfactorily inform them about disease management. These findings are similar to global reports in which those affected by PCOS describe not adequately being informed by their health care practitioner, or were not referred to relevant sources of information at diagnosis, leading to overall dissatisfaction with their health care [2, 30, 31]. In other studies, it was also reported that patients were not satisfied with information provided or available on medical therapies, lifestyle management, long-term complications, and potential infertility [46]. In our study and in other reports, those with PCOS are scarcely informed about cardiometabolic risk factors and long-term morbidity risk including dyslipidemia, insulin resistance, pre-diabetes, diabetes and cardiovascular disease [71–73]. For example, a respondent stated her frustration with the lack of information and adequate help as follows: “*I am exhausted from constantly fighting with my doctors to help me manage my PCOS diagnosis. At this point, I would rather forgo everything I have accomplished and let this horrible chronic condition progress to a more serious version (ie developing diabetes) so maybe I could get help.*”

International recommendations for the management and diagnosis of PCOS indicate that educational resources for patients should be multimodal, comprehensive, evidence-based, and highlight peer support groups [3, 38]. As well, biopsychosocial dimensions such as biological, psychological, and social factors and their interactions should be considered in understanding PCOS illness and its health care delivery. Behavioral and lifestyle interventions need to be guided by expert knowledge and evidence-informed options. Frustration about a lack of information and expert advice can lead to distrust in the health care system and precipitate poor relationships between patients and health care professionals [74]. Establishing a partnership between the health professional and the individual is essential for targeted and appropriate treatment decisions and optimal management of PCOS symptoms and co-morbidities [3, 38].

### Self-sourcing of information and self-advocacy

Respondents reported frequently (82%) relying on internet resources and peer support groups to inform themselves on management options and comorbidity risks in PCOS. In addition, respondents reported the need to advocate for themselves and to request referrals to specialist care. These findings are consistent with another Canadian study which reported a reliance on self-education and self-advocacy by respondents in their PCOS management [48]. A study by Hoyos et al. found that almost all (98%) of survey respondents searched for information about PCOS on the internet [28]. Whilst online support groups are beneficial for peer support, internet and social media forums may not always provide validated and reliable sources of evidence-based information and this information may mis-inform those with PCOS [75, 76].

### Recommendations for health care improvement suggested by survey respondents

Our survey results provide evidence for the need to develop better pathways to care for patients at-risk or diagnosed with PCOS in the areas of diagnosis, management, and preventative health care strategies to improve overall health. This would include referral, ongoing care and access to specialist health care services for people living with PCOS in Alberta and Canada.

One of the primary concerns of respondents was the lack of expert information on the reproductive-endocrine physiology of PCOS, on management options, and on PCOS impact as a risk factor for co-morbidities. Respondents attributed the delay in diagnosis to family physicians’ and general practitioners’ lack of expertise in diagnosis and management of PCOS *(“More doctors need to know about PCOS! They need to know what blood work needs to be done, from what I've seen, they don't”*) and the lack in referral to experts such as endocrinologists and Ob/Gyn specialists (“*I think that there should be faster referrals to OB's or GYN'S*”). Early diagnosis is essential for proper initiation of an individually tailored management plan. Improvement in diagnosis and initial management may be achieved through increased awareness of PCOS and an enhanced inclusion of diagnosis and management in the education of medical students, general practitioners and allied health personnel (“*Maybe more training and compassion from primary care physicians so I don't want to avoid going*”). In addition, it is crucial for women with PCOS to be assessed for need of referral after diagnosis to a range of health care professionals in order to initiate effective intervention early and reduce risk of developing morbidities such as diabetes. In addition, timely and informative care and evidence-based knowledge about endocrine-metabolic disorders and risk of cardiovascular disease or diabetes, would help to engage and prepare respondents in managing their health care (“*I would have appreciated more accurate and useful information at a young age*”).

Many of the concerns at follow-up among our survey respondents are similar to those at diagnosis, for example the lack of adequate information and continuous care with regard to follow-up and referrals to experts. Considering that there is currently no cure for PCOS, providing continuous medical care and diet-lifestyle advice is necessary to manage symptoms and development of co-morbidities. Because of the diversity of physical and mental symptoms, the health care of individuals with PCOS should include a variety of experts such as endocrinologists, obstetrician/gynecologists, dietician/nutritionists, physiotherapists/exercise specialists, psychologists/psychiatrists and other additional allied health care workers as requested (“*Refer to specialist that can provide adequate resources and information on PCOS*”). Information from the international evidence-based guidelines emphasize the importance of accessible evidence-based information and multidisciplinary support in the management of PCOS [3].

An optimal solution would be to establish specialized multidisciplinary PCOS clinics as suggested by respondents (“*Have all the help needed for PCOS in one clinic; endocrinologist, gynecologist, naturopath, dietitian, acupuncture, psychologist, hair removal clinic, and counseling*. “and “*I hope there will be a center where this condition is addressed so we could have better chances of results being processed on a timely basis. “*). A specialized PCOS clinic would provide wholistic health care and access to a variety of appropriate health care professionals including clinicians and allied health care workers in one location, similar to interdisciplinary clinics established for diabetes and obesity.

Our survey has found that those affected with PCOS advocate for themselves and frequently self-manage their disease. This includes consulting the internet, requesting proper information and seeking expert advice when necessary. Respondents stated that they want to be involved in their care; they want to be taken seriously when discussing their symptoms, their pain, and their goals for management (“*Take our pain seriously. Help us find solutions and not band aid.”*). They want to have their questions answered and weigh their PCOS management options. PCOS requires a unique and individualized health care approach which can only be achieved if clinicians work in partnership with patients. Detailed information on the course of the disease and treatment options should be provided by health care personnel without the need to self-source information from the internet.

Survey respondents also suggested those with experience living with PCOS should be included as partners in PCOS-related studies (“*I'm interested in the research coming out about PCOS, which is why I was interested in the study*”). Studies are needed to deepen the understanding of symptoms and evaluate the efficacy and safety of the different treatment options, and PCOS patients themselves have the know-how to advise on research directions and interpret outcomes. Knowledge of personal experience with PCOS is needed to help others with their health care journey, provide tailored medical care and to prevent adverse outcomes.

**Fig. 2:**
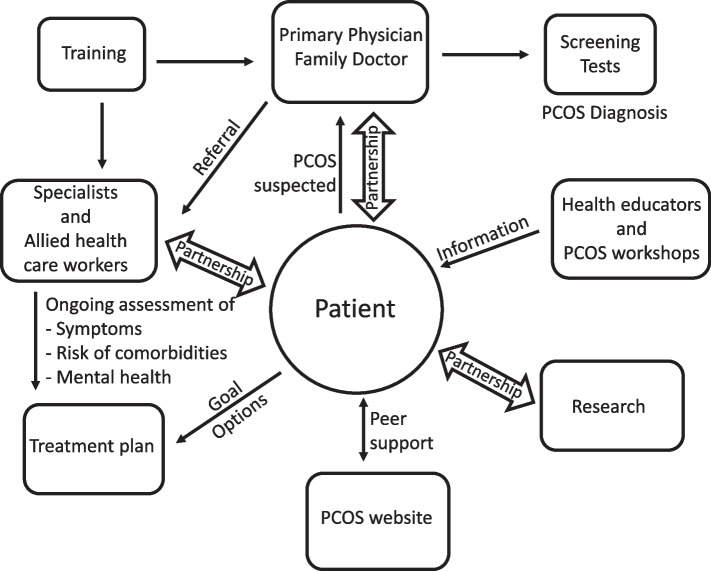
Patient-centered multidisciplinary pathways to care model for PCOS

Based on survey respondents’ recommendations to improve health care for those with PCOS, we have a proposed a patient centered model of care, outlined in Fig. 2. Such care could be provided at multidisciplinary, specialized PCOS clinics and/or involve better co-ordination and resource allocation of existing services.

### Limitations and strengths of the study

The survey is based on a published questionnaire used in those with PCOS [2, 30] and expanded to include more questions, and was adapted to the Canadian health care system. Therefore, the survey results may not be generalizable to other countries and/or health care systems. Our survey was adapted in collaboration with patient-partners and health care professionals that diagnose those at risk for PCOS and manage those affected by PCOS. A pilot survey was tested with a focus on the Alberta health care system which may be a limitation in applying to other countries or provincial health care systems. The sample size was small for a population-based survey and multi-variable analyses. However, the sample was sufficient to gain insight into health concerns and for a qualitative thematic analysis of answers to open questions. A limitation is that respondents may have recall bias depending on the time frame of first symptoms, diagnosis and clinician visits. The survey is based on a self-reported diagnosis of PCOS and was not verified with medical records due to the anonymous nature of the survey. However, the anonymity of the survey is an advantage for respondents to safely and openly share their perceptions and experience in the health care system. We also cannot exclude the possibility of a sample bias towards those who were more dissatisfied with their health care experience. The questionnaire was only provided in the English language, further limiting the collection of data from a more diverse population of those affected by PCOS in Alberta and Canada. Our study participants were predominately Caucasian (70%) and 20% were immigrants to Canada, therefore data may not reflect the diverse ethnic groups including indigenous groups in Canada, and future studies could be aimed to address this limitation. The internet-based delivery of the survey and advertising of the survey through social media may have also reduced the diversity of survey respondents due to limited access. In this study, we did not investigate the relationship between intersectional factors and how these may impact health and health care experience including gender, ethnicity, socioeconomic status, family history, family dynamics, work and childcare [77, 78].

The strength of the survey lies in the comprehensive questions encompassing all steps of the PCOS health care journey from first symptom experience via diagnosis and follow-ups to lifetime management of the disease. The survey provided sufficient questions with yes/no answers and answers from a variety of drop-down choices; as well it allowed respondents to voice their opinion in open questions; together, this yielded an overall image of the perceptions and experience of those affected by PCOS in the health care system in Alberta, Canada. We also provided opportunity for reflection on barriers and challenges in the health care system and respondents were able to consider suggestions for improvements in pathways to care for those affected by PCOS.

## Conclusion

At present in Canada, and especially in Alberta, timely diagnosis, information and resources, increased options for symptom management, continuous support and multidisciplinary care are not currently adequate for those affected by PCOS. Capturing the perception and experiences of those living with PCOS provided a patient-focussed insight and suggestions for improvements in health care, including education of physicians and health care providers, timely diagnosis, referrals to specialists, resource development for patient education, and involvement of PCOS patients as partners in their health care and in PCOS-related research. Optimal support and care for diverse PCOS symptoms and risk of co-morbidities could be serviced by establishing specialized-multidisciplinary PCOS clinics. These findings are a first step to understanding the barriers and challenges faced by those with PCOS. The future direction on this work is translating these findings in raising awareness of PCOS and the development of pathways to care that includes health care provider and patient education to improve the health and health care experience of those affected by PCOS.

### Supplementary Information

**Additional file 1: Supplementary Table 1.** Socioeconomic Attributes of Survey Respondents.

## Data Availability

Complete survey data pertaining to the current study are available from the corresponding author in accordance with appropriate data use agreements.
