# Supplementary material for: Challenges in diagnosis and health care in polycystic ovary syndrome in Canada: a patient view to improve health care
Source: BMC Womens Health. 2023 Nov 4;23:569. doi: 10.1186/s12905-023-02732-2 (PMC10625259; doi:10.1186/s12905-023-02732-2)
Supplement: Supplementary file 1 — Additional file 1: Supplementary Table 1. Socioeconomic Attributes of Survey Respondents. [file 12905_2023_2732_MOESM1_ESM.docx]

**Supplementary Table 1: Socioeconomic Attributes of Survey Respondents**

| **Participant attributes for N = 194** | | **N [%]** |
| --- | --- | --- |
| ***Education level*** | Some high school  High school diploma  Certificate  Bachelors  Postgraduate degree | 4 [2.1]  39 [20.1]  64 [33.0]  64 [33.0]  21 [10.8] |
| ***Annual household income*** | <10,000  10,000-30,000  30,000-60,000  60,000-80,000  80,000-120,000  120,000-160,000  160,000-200,000  >200,000  Not reported | 5 [2.6]  29 [14.9]  44 [22.7]  45 [23.2]  28 [14.4]  8 [4.1]  8 [4.1]  1 [0.5]  14 [7.2] |
| ***Work status*** | Not employed  Self employed  Unpaid work  Part-time/contract  Full time work  Part-time study  Full-time study | 25 [12.9]  17 [8.8]  3 [1.5]  31 [16.0]  112 [57.7]  7 [3.6]  20 [10.3] |
| ***Children*** | none  1  2  3  4 or more | 116 [59.8]  26 [13.4]  27 [13.9]  18 [9.3]  6 [3.1] |
| ***Living condition*** | Alone  With partner +/- dependent  With parents  With roommate(s) | 16 [8.2]  147 [75.8]  18 [9.3]  12 [6.2] |
| ***Community population*** | Rural ^a^  Small population centre  Medium population centre  Large population centre  Very large population centre | 20 [10.3]  20 [10.3]  41 [21.1]  21 [10.8]  89 [45.9] |

^a^ with 2 reporting living less than 10 km from a larger population centre, 13 living within 10-50 km and 4 living 50 – 100 km from a larger population centre
